# 1105. Clinical outcomes in non-pregnant adults aged ≥18 years hospitalized with laboratory-confirmed RSV infection, 12 U.S. states, October 2014–April 2022

**DOI:** 10.1093/ofid/ofad500.078

**Published:** 2023-11-27

**Authors:** Michael Melgar, Michael Whitaker, Pam Daily Kirley, Nisha B Alden, Daewi Kim, Kyle P Openo, Alicia Brooks, Lauren Leegwater, Erica Bye, Francesca Pacheco, Grant Barney, Kevin Popham, Melissa Sutton, H Keipp Talbot, Ryan Chatelain, Fiona P Havers

**Affiliations:** Centers for Disease Control and Prevention, Atlanta, Georgia; CDC, Marietta, Georgia; California Emerging Infections Program, Oakland, California; Colorado Department of Public Health and Environment, Denver, Colorado; Yale CT Emerging Infections Program, New Haven, Connecticut; Georgia Emerging Infections Program and Atlanta VA Medical Center, Decatur, GA; Maryland Department of Health, Baltimore, Maryland; Michigan Department of Health & Human Services, Grand Rapids, Michigan; Minnesota Department of Health, Saint Paul, Minnesota; University of New Mexico Health Sciences Center, Albuquerque, New Mexico; New York State Department of Health, Albany, New York; Universirty of Rochester, Rochester, New York; Oregon Health Authority, Portland, Oregon; Vanderbilt University Medical Center, Nashville, Tennessee; Salt Lake County Health Department, Salt Lake City, Utah; CDC, Marietta, Georgia

## Abstract

**Background:**

Respiratory syncytial virus (RSV) is an important cause of respiratory illness and hospitalization in older adults and adults with certain underlying medical conditions. With novel RSV vaccines in development, it is critical to identify adults at increased risk of severe illness.

**Methods:**

Population-based surveillance was conducted through the RSV Hospitalization Surveillance Network (RSV-NET) over 8 seasons (2014–2022) across 75 counties in 12 states. We included non-pregnant adults (≥ 18 years) residing in the RSV-NET catchment area who were hospitalized with laboratory-confirmed RSV infection (clinician-directed testing) during each season (October–April, except 2020–2021, which spanned October 2020–September 2021). Demographic and outcomes data (all seasons) and underlying medical conditions (October 2014–April 2018) were abstracted from medical records. We calculated percentages of adults with intensive care unit (ICU) admission, mechanical ventilation (MV), and in-hospital death, stratified by demographic characteristics. We calculated age-adjusted percentages with these outcomes, stratified by underlying conditions.

**Results:**

We identified 13,080 RSV-associated hospitalizations among adults; 61.9% were aged ≥ 65 years (**Table 1**). ICU admission was recorded in 18.6%, MV in 7.2%, and in-hospital death in 4.2%. Most in-hospital deaths (54.1%) occurred among adults aged ≥ 75 years. Among adults 18–49 years, immune compromise (34.8%) and asthma (29.8%) were the most frequent underlying conditions (**Figure**). Adults with non-asthma chronic lung disease (CLD) and with cardiovascular diseases had the highest age-adjusted percentages of in-hospital death (**Table 2**). Adults with non-asthma CLD had the highest age-adjusted percentages of ICU admission and MV.

**Figure:**
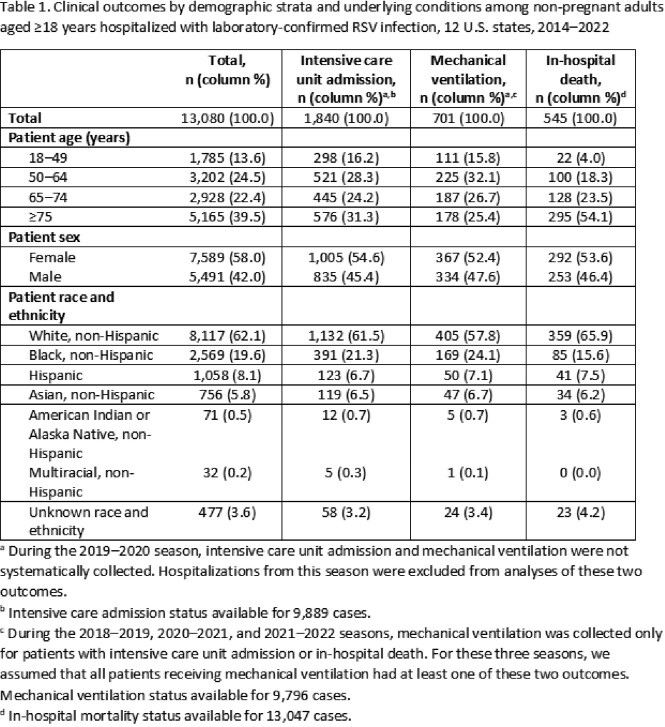

**Figure:**
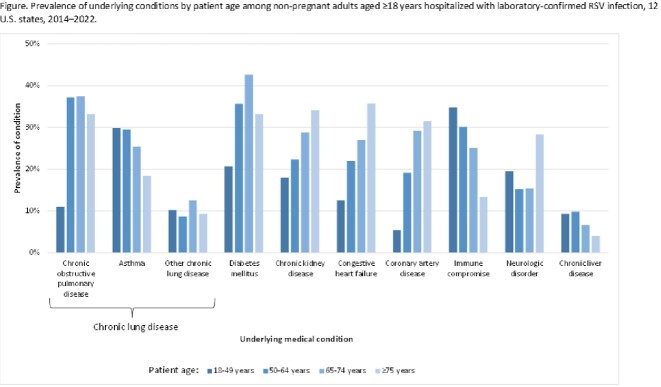

**Figure:**
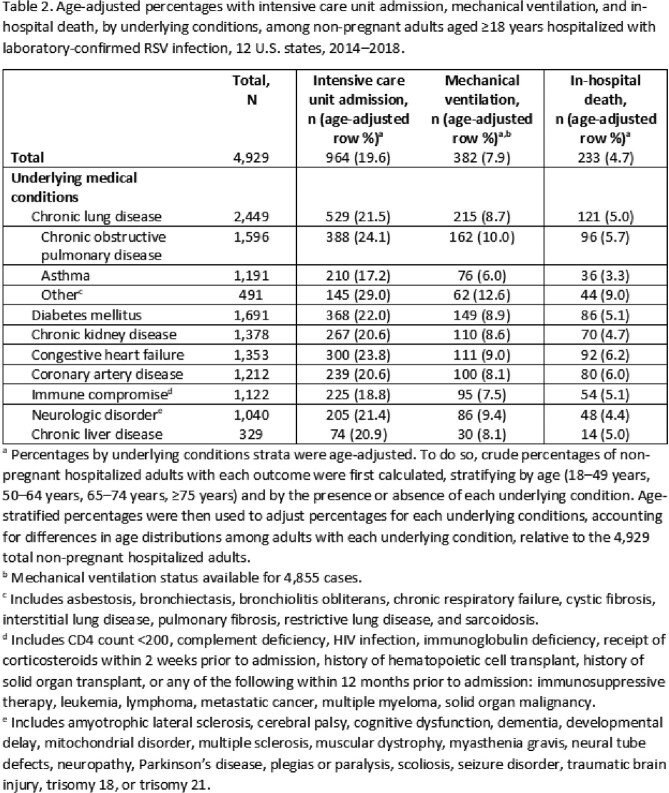

**Conclusion:**

Among adults with RSV-associated hospitalization, older age and cardiopulmonary conditions were associated with severe illness. Hospitalized young adults more likely had immune compromise or asthma. This study was limited by clinician-driven testing, which likely under-detected RSV-associated hospitalizations. Older adults and adults with cardiopulmonary and immune compromising conditions may benefit from RSV vaccination when licensed products become available.

**Disclosures:**

**All Authors**: No reported disclosures

